# Peanut witches' broom (PnWB) phytoplasma-mediated leafy flower symptoms and abnormal vascular bundles development

**DOI:** 10.1080/15592324.2015.1107690

**Published:** 2015-10-22

**Authors:** Chi-Te Liu, Hsin-Mei Huang, Syuan-Fei Hong, Ling-Long Kuo-Huang, Chiao-Yin Yang, Yen-Yu Lin, Chan-Pin Lin, Shih-Shun Lin

**Affiliations:** 1 Institue of Biotechnology; National Taiwan University; Taipei, Taiwan; 2 Agricultural Biotechnology Research Center; Academia Sinica; Taipei, Taiwan; 3 Center of Biotechnology; National Taiwan University; Taipei, Taiwan; 4 Department of Life Science; National Taiwan University; Taipei, Taiwan; 5 Department of Plant Pathology and Microbiology; National Taiwan University; Taipei, Taiwan

**Keywords:** internal phloem, leafy flower, peanut witches', broom phytoplasma, vascular bundle, 4′,6′-diamidino-2-phenylindole staining

## Abstract

The peanut witches' broom (PnWB) phytoplasma causes virescence symptoms such as phyllody (leafy flower) in infected peanuts. However, the obligate nature of phytoplasma limits the study of host-pathogen interactions, and the detailed anatomy of PnWB-infected plants has yet to be reported. Here, we demonstrate that 4′,6′-diamidino-2-phenylindole (DAPI) staining can be used to track PnWB infection. The DAPI-stained phytoplasma cells were observed in phloem/internal phloem tissues, and changes in vascular bundle morphology, including increasing pith rays and thinner cell walls in the xylem, were found. We also discerned the cell types comprising PnWB in infected sieve tube members. These results suggest that the presence of PnWB in phloem tissue facilitates the transmission of phytoplasma via sap-feeding insect vectors. In addition, PnWB in sieve tube members and changes in vascular bundle morphology might strongly promote the ability of phytoplasmas to assimilate nutrients. These data will help further an understanding of the obligate life cycle and host-pathogen interactions of phytoplasma.

Phytoplasmas, bacteria belonging to the class *Mollicutes* that lack a cell wall, are obligate bacteria of phloem tissue. Phytoplasma requires a sap-feeding insect vector for transmission or the grafting of infected branches onto healthy plants for infection. Therefore, the obligate nature constitutes a bottleneck for research investigating phytoplasma. Peanut witches' broom (PnWB) phytoplasma, of the genus *Ca. phytoplasma*, was initially collected from a naturally infected peanut field on the Penghu Islands of Taiwan in 1985 (**Fig. 1A**), designated herein as the PnWB PH isolate.^1^ According to the molecular phylogeny inferred from the 16S rDNA sequence, PnWB was placed within the third clade in the genus, belonging to the peanut witches' broom group (16SrII).^2^

**Figure 1.**
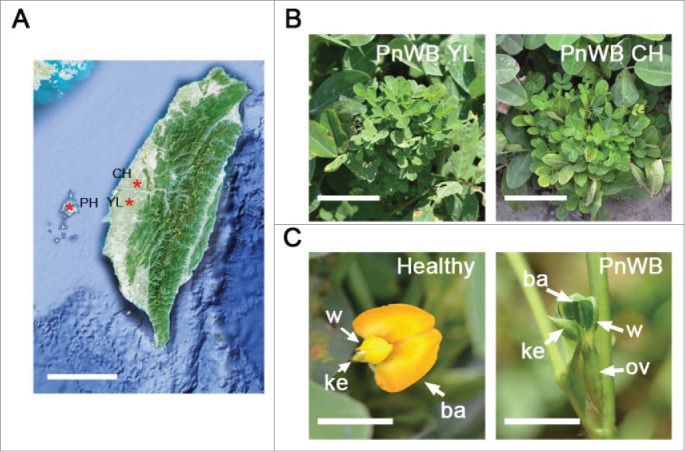
Peanut witches' broom (PnWB)-infected peanut plants were collected from peanut fields of Taiwan.(A) The geographic locations of PnWB isolates in Taiwan are indicated by red asterisks. PH refers to the Penghu Island; CH refers to the Changhua area; YL refers to the Yunlin area. Bar, 100 km. (B) Witches' broom symptoms of PnWB-infected peanuts collected from the Yunlin and Changhua areas. PnWB CH refers to PnWB-infected peanuts collected from Changhua. PnWB YL refers to PnWB-infected peanuts collected from Yunlin. Bar, 1 cm. (C) Healthy (left panel) and PnWB-infected (right panel) peanut flowers. ba: banner; w: wings; ke: keel; ov: ovary. Bar, 0.2 cm.

PnWB causes virescence symptoms in peanut plants.^1^ Based on such symptoms, we collected 2 phytoplasma isolates from natural peanut fields in the Changhua and Yunlin areas of Taiwan, which have a geographic relationship (<100 km) with Penghu Island (**Fig. 1A, and B**). The 16S rRNA and *Phyllody symptoms1* (*PHYL1*) effector ^3^ sequence data indicated that the 2 isolates have 100% nucleotide identity with the PnWB PH isolate, suggesting that these 2 isolates belong to PnWB group (designated as the PnWB YL and CH isolates, respectively).

Phyllody (referred to herein as leafy flower) is a well-known symptom on phytoplasma-infected plants. Indeed, leafy flower symptoms were observed on PnWB CH-infected peanut plants (**Fig. 1C**); healthy flowers displayed yellow and normal banner, wings, and keel structures (**Fig. 1C**, left panel), whereas PnWB-infected flowers exhibited green, leafy structures (**Fig. 1C**, right panel). The phytoplasma PHYL1 effector triggers the proteasomal degradation of flowering MADS-box transcription factors (e.g., *APETALA1, SEPALIATA3*, and *CAULIFLOWER*) and interferes with the regulation of microRNA396 (miR396)-*SHORT VEGETATIVE PHASE*, resulting in leafy flower formation.^3-5^ A whole-transcriptome analysis demonstrated that several genes expressed during reproductive or vegetative stages were misregulated, resulting in switches in plant phase transition.^6^

The morphology of vascular bundles in PnWB-infected *Catharanthus roseus*, which is widely used as a model plant for phytoplasma infection, was investigated. On cross-sections, a healthy stem displayed sequentially, a xylem (light blue color), a cambium zone, and phloem (**Fig. 2A**, left panel), whereas an abnormal vascular bundle was observed in PnWB-infected stems (**Fig. 2A**, right panel). In PnWB-infected plants, many pith rays had formed (green arrowheads), dividing the xylem zone into several small fragments (**Fig. 2A**, right panel). In general, pith rays play a role in horizontal nutritional transport between pith and phloem tissues. Moreover, the cell walls of the PnWB-infected xylem cells were thinner than those of healthy xylem cells (**Fig. 2A**), suggesting that the nutritional support of host for the phytoplasma infection is more precedent than the provision of water. Indeed, the phytoplasma has a minimal genome size (˜0.5 Mb) of approximately 500 genes, and it lacks many of the genes that are important for cell metabolic processes, such as nucleotide synthesis and ATP biogenesis.^2,7^ Besides, the *SUCROSE ISOZYME SH1* gene is induced in phytoplasma-infected companion cells.^8^ Therefore, the phytoplasma must strongly rely on nutrient uptake from the host and increasing the number of pith rays in infected plants might represent one underlying strategy.

**Figure 2.**
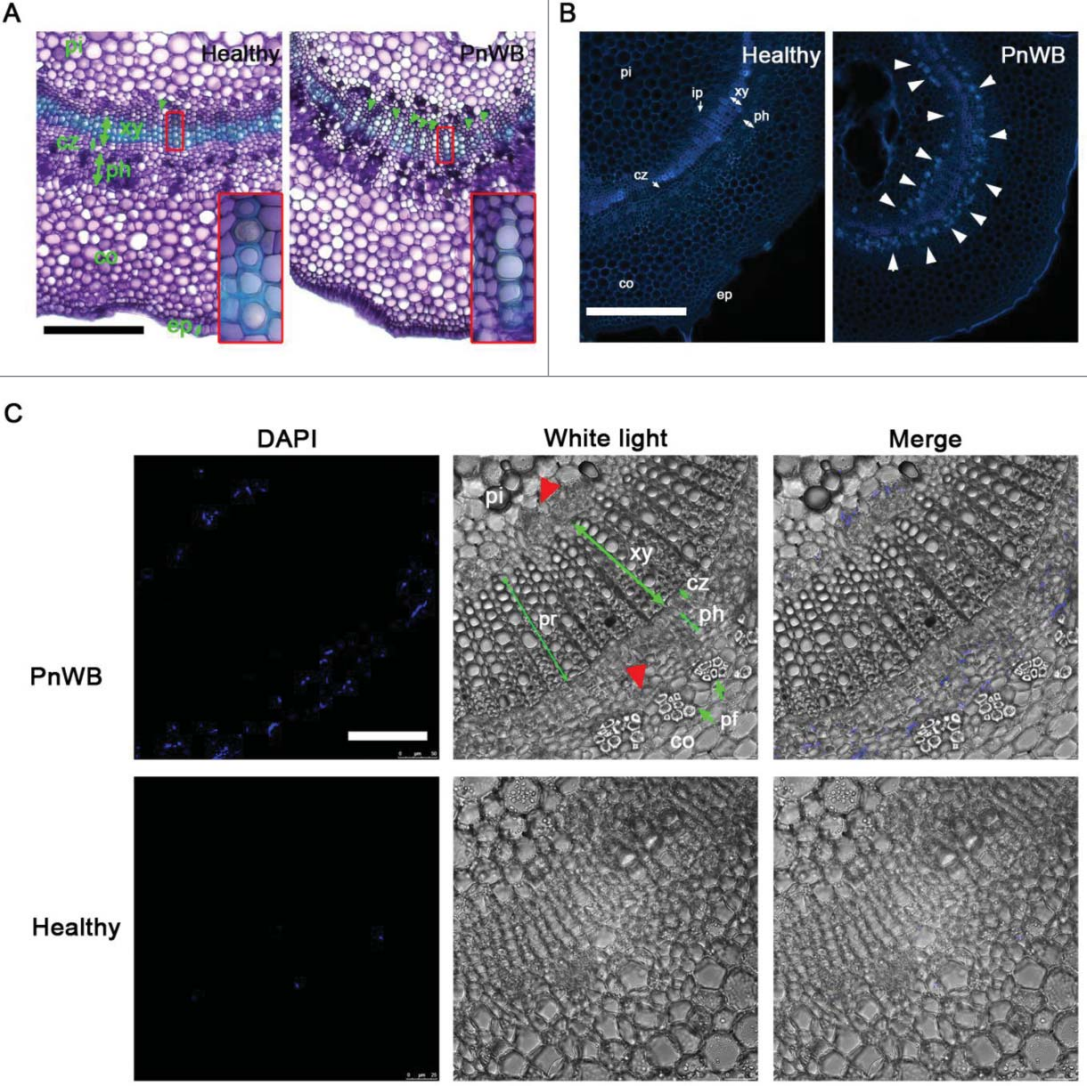
Peanut witches' broom (PnWB) infection alters vascular bundle development.(A) Transverse sections of healthy (left panel) and PnWB-infected (right panel) *C. roseus* stems stained with toluidine blue O. Green arrowheads indicate pith rays. The enlarged photograph shows the xylem (xy). Bars, 0.2 mm. (B) Transverse section of healthy (left panel) and PnWB-infected (right panel) *C. roseus* stems stained with 4′,6′-diamidino-2-phenylindole (DAPI). White arrowheads indicate PnWB-infected cells. Bars, 0.05 mm. (C) Cross-section of PnWB-infected stem tissue (upper panel) stained with DAPI. Red arrowheads indicate PnWB-infected cells. Cross-section of healthy stem tissue (lower panel), which was used as a negative control. Pith (pi), pith ray (pr), xylem (xy), cambium zone (cz), phloem (ph), phloem fiber (pf), and cortex (co) are indicated by green arrows. Bar, 100 µm.

Next, healthy and PnWB-infected *C. roseus* tissues were stained with 4′,6′-diamidino-2-phenylindole (DAPI), and cross-sections were observed using a fluorescence microscope. Many reports have demonstrated that phytoplasmas reside in sieve tube members,^7,9^ which lack nuclei. Thus, the DAPI-stained chromosome of the phytoplasma provides a good tracking method to observe its localization. During UV excitation, the autofluorescence of the *C. roseus* tissues clearly revealed the structures of the pith, xylem, cambium zone, phloem, cortex, and epidermis in healthy stems (**Fig. 2B**, panel left). Interestingly, many additional fluorescent spots (white arrowheads) were showed at both edges of the vascular bundle (**Fig. 2B**, right panel). We further investigated the DAPI-stained vascular bundle region using a highly magnified (630×) image obtained by confocal microscopy. Very few fluorescent DAPI spots were observed in phloem of healthy tissues, suggesting an absence of cell nuclei of sieve tube members (**Fig. 2C**, lower panel). In contrast, many irregular fluorescence DAPI spots were observed in the phloem and the region of internal phloem (the region between the pith and xylem zone) (**Fig. 2C**, upper panel). We assumed that the phytoplasma was stained with DAPI and therefore present in phloem tissues. Herein, we, for the first time, demonstrate replication of phytoplasma in the internal phloem of *C. roseus* (**Fig. 2C**, upper panel). Monoterpene indole alkaloid (MIA) biosynthesis occurs in the internal and primary phloem of *C. roseus*,^10,11^ and in future studies, it will be interesting to determine whether the phytoplasma in these phloem tissues alters MIA biosynthesis.

Using image that were magnified 1000×, the DAPI-stained phytoplasma cells were localized in sieve tube members; in **Figure 3A**, red arrowheads indicate PnWB-infected sieve tube members, which also exhibit a condensed cytoplasm (**Fig. 3A**). We further observed phytoplasma cells inside sieve tube members by examining ultrathin sections of phloem cells using transmission electron microscopy (**Fig. 3B**). No phytoplasma cells were observed in ultrathin sections of sieve tube members of healthy *C. roseus* (**Fig. 3B**, panel i), whereas the PnWB-infected sieve tube members were filled with phytoplasma and impurities (**Fig. 3B**, panel ii). Such impurities have been suggested to be P-proteins of the sieve tube or pathogen-related proteins.^12-14^ The PnWB cells are 50×50 to 890×700 nm in diameter, depending on the segment angle (**Fig. 3B**, panel ii), and exhibit an elliptical, circular and amorphous morphology (**Fig. 3C**, panel ii, iii, and iv). DNA fibrils (DF) with net-like structures in the phytoplasma cells and ribosomes (R) can also be observed (**Fig. 3C**, panel iii, and iv). The membrane is less than 27 nm (**Fig. 3C**, panel iv). Smaller bodies (SB) with electron-dense contents can also be observed in infected sieve tubes, and these were considered to be pathogen-related proteins (**Fig. 3C**, panel ii, and iv).

**Figure 3.**
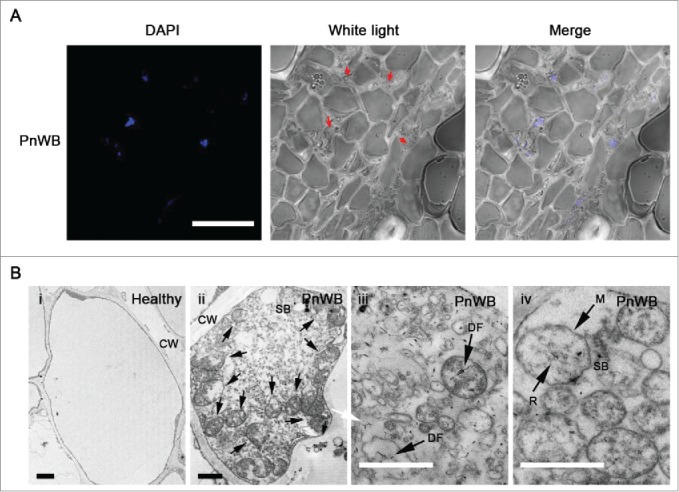
Microscopic observation of peanut witches' broom (PnWB)-infected stem. (A) Detection of PnWB-infected cells by confocal microscopy. The stem sections were stained with 4′,6′-diamidino-2-phenylindole (DAPI). Red arrowheads indicate infected sieve tube members. Bar, 25 µm. (B) Ultrathin sections of sieve tube members from healthy (panel i) and PnWB-infected (panel ii) *Catharanthus roseus* plants. Red arrowheads indicate PnWB phytoplasma cells. The pleomorphic morphology of the PnWB cell is shown. DNA fibrils (DF) (panel iii), and ribosomes (R) and cell membrane (M) of PnWB (panel iv). CW, cell wall of the host cell. SB, smaller bodies. Bars, 1 µm.

Here, we demonstrate leafy flower symptoms on PnWB-infected peanut flowers and the ability to track PnWB infection by DAPI staining. Instead of leafy flowers, the morphology of the vascular bundle was altered to enhance nutrition for supporting PnWB infection. We also observed the PnWB cell type in the sieve tubes of *C. roseus* and phloem/internal phloem tissues were found to be the site of infection. Such discoveries will help to increase our knowledge regarding the PnWB life cycle and host-PnWB interactions.
